# Myocardial Radiomics Texture Features Associated with Increased Coronary Calcium Score—First Results of a Photon-Counting CT

**DOI:** 10.3390/diagnostics12071663

**Published:** 2022-07-08

**Authors:** Isabelle Ayx, Hishan Tharmaseelan, Alexander Hertel, Dominik Nörenberg, Daniel Overhoff, Lukas T. Rotkopf, Philipp Riffel, Stefan O. Schoenberg, Matthias F. Froelich

**Affiliations:** 1 Department of Radiology and Nuclear Medicine, University Medical Center Mannheim, Heidelberg University, Theodor-Kutzer-Ufer 1-3, 68167 Mannheim, Germany; hishan.tharmaseelan@medma.uni-heidelberg.de (H.T.); alexander.hertel@medma.uni-heidelberg.de (A.H.); dominik.noerenberg@medma.uni-heidelberg.de (D.N.); daniel.overhoff@umm.de (D.O.); philipp.riffel@umm.de (P.R.); stefan.schoenberg@umm.de (S.O.S.); matthias.froelich@medma.uni-heidelberg.de (M.F.F.); 2 Department of Diagnostic and Interventional Radiology and Neuroradiology, Bundeswehr Central Hospital Koblenz, Rübenacher Straße 170, 56072 Koblenz, Germany; 3 Department of Radiology, German Cancer Research Center, Im Neuenheimer Feld 280, 69120 Heidelberg, Germany; l.rotkopf@dkfz-heidelberg.de

**Keywords:** photon-counting computed tomography, coronary artery calcium score, radiomics, texture analysis

## Abstract

The coronary artery calcium score is an independent risk factor of the development of adverse cardiac events. The severity of coronary artery calcification may influence the myocardial texture. Due to higher spatial resolution and signal-to-noise ratio, new CT technologies such as PCCT may improve the detection of texture alterations depending on the severity of coronary artery calcification. In this retrospective, single-center, IRB-approved study, left ventricular myocardium was segmented and radiomics features were extracted using pyradiomics. The mean and standard deviation with the Pearson correlation coefficient for correlations of features were calculated and visualized as boxplots and heatmaps. Random forest feature selection was performed. Thirty patients (26.7% women, median age 58 years) were enrolled in the study. Patients were divided into two subgroups depending on the severity of coronary artery calcification (Agatston score 0 and Agatston score ≥ 100). Through random forest feature selection, a set of four higher-order features could be defined to discriminate myocardial texture between the two groups. When including the additional Agatston 1–99 groups as a validation, a severity-associated change in feature intensity was detected. A subset of radiomics features texture alterations of the left ventricular myocardium was associated with the severity of coronary artery calcification estimated by the Agatston score.

## 1. Introduction

The coronary artery calcium score (CACS) is an established risk predictor of adverse coronary events such as myocardial infarction and cardiac death [1,2]. Through the CACS, the individual burden of subclinical atherosclerosis of coronary arteries can be determined. However, the significance of CACS extends far beyond purely estimating the cardiac risk; it is also associated with other disease patterns such as cancer and dementia, partly due to underlying similar risk factors such as tobacco use, diabetes, and hypertension [3]. The Agatston score is used in daily practice to estimate the extent of coronary artery calcification not only as a total but also differentiated to the individual main vessels [4]. Using the Agatston score, a risk profile can be created that indicates a cardiac age in relation to the biological age and, depending on the extent of the calcifications (and, thus, the level of the score), a probability for the development of an obstructive coronary artery disease event in the next 10 years [5]. The cut-off value of 100 was outlined as a reference point, as calcium scores greater than 100 were associated with most coronary events such as myocardial infarction or death from coronary artery disease [6]. As cardiovascular diseases remain the number one cause of death worldwide [7], the importance of further diagnostic tools in this field has been recognized by medical societies, leading to a readjustment of guidelines and recommendations for cardiac CT [8], even with the knowledge of the limitation in cases of severe artery calcification due to the blooming artifacts of calcified plaque that occur due to limited spatial resolution [9]. This was even further emphasized by outlining the lower frequency of major-procedure-related complications in patients with stable chest pain and intermediate pretest probability of coronary artery disease who underwent initial CT instead of initial invasive coronary angiography, as outlined by the DISCHARGE trial [10].

Apart from risk estimations based on CACS, imaging of the myocardium, followed by assessment of myocardial disease, with magnetic resonance imaging (MRI) is a further important clinical parameter in these patients. A recent study revealed CACS as an independent predictor of unrecognized myocardial infarction on MRI [11]. In the context of MRI, texture analysis with radiomics parameters has been established as an additional quantitative clinical marker of the myocardium [12,13,14]. Radiomics is a technique used to extract numerous features from an area of interest in a radiologic image to create datasets of hundreds of parameters [15,16]. Recently, the first feasibility studies have employed radiomics for analysis of myocardial fibrosis [17], quantification of coronary plaques [18], and perivascular fat [19] on computed tomography. However, the effect of increased CACS on myocardial texture features on CT is unclear. Coronary artery sclerosis may have a chronic impact on myocardial perfusion, possibly resulting in myocardial fibrosis. Myocardial fibrosis was, until now, mainly investigated in cardiac MRI [12] due to the need for optimal spatial resolution and signal-to-noise ratio [20,21,22].

The implementation of photon-counting computed tomography (PCCT) has the potential to overcome this limitation. In contrast to conventional energy-integrating detectors (EIDs), PCCT allows every photon that hits the detector element to be directly converted into an electric pulse. Due to smaller detector elements and using a threshold for electric noise, PCCT has higher spatial resolution as well as a better signal-to-noise ratio [23,24].

The aim of this pilot study was to investigate whether myocardial texture changes not visible to the human eye can be identified by texture analysis depending on the severity of coronary artery calcification with PCCT.

## 2. Materials and Methods

### 2.1. Study Design

For this retrospective single-center study, patients with clinically indicated electrocardiography (ECG) gated non-contrast- and contrast-enhanced cardiac CT were enrolled between December 2021 and February 2022. All patients were examined using a clinically approved photon-counting CT system. Patients with suspected or known CAD were included. Patients were excluded in the case of severe image artifacts (*n* = 4) or in the case of previous cardiac stent implantation (*n* = 1). Additionally, patients were excluded in the case of known cardiomyopathy, history of myocardial infarction, or visible myocardial damage (*n* = 4). All investigations were conducted according to the Declaration of Helsinki. The study had institutional review board and local ethics committee approval (ID 2021-659).

### 2.2. Patient Collective

Based on inclusion and exclusion criteria, a total of 30 CT scans of patients were included in this study. In total, 30 patients (22 men, 8 women; mean age 58 years, range: 21–80 years; all *p* > 0.05) were selected. The patient and scan characteristics are summarized in Table 1.

### 2.3. Chest CT Imaging

All 30 patients were scanned on a first-generation, whole-body, dual-source PCCT system (NAEOTOM Alpha; Siemens Healthcare GmbH, Forchheim, Germany) using a prospective ECG-gated sequential mode with a tube voltage of 120 kV and automatic dose modulation with a CARE keV BQ setting of 64, and gantry rotation time was 0.25 s. In the absence of contraindications and in correlation to heart rate, patients intravenously received 5–10 mg of ß-blockers to lower heart rates to less than 65 beats/min. First, all patients underwent a non-contrast-enhanced cardiac CT (2 mm slice thickness) for the evaluation of coronary artery calcification by estimation of the Agatston score. The contrast-enhanced scan of the coronary arteries was performed using 80 mL of iodine contrast (Imeron 400, Bracco Imaging Deutschland GmbH, Konstanz, Germany) followed by a 20 mL saline chaser (NaCl 0.9%) with a weight-based flow rate of 5–6 mL/s via antecubital venous access. Bolus tracking was used to trigger the start of coronary CTA by placing a region of interest (ROI) in the descending thoracic aorta (threshold 140 HU at 90 kV).

### 2.4. Chest CT Imaging Analysis

**Figure 1:**
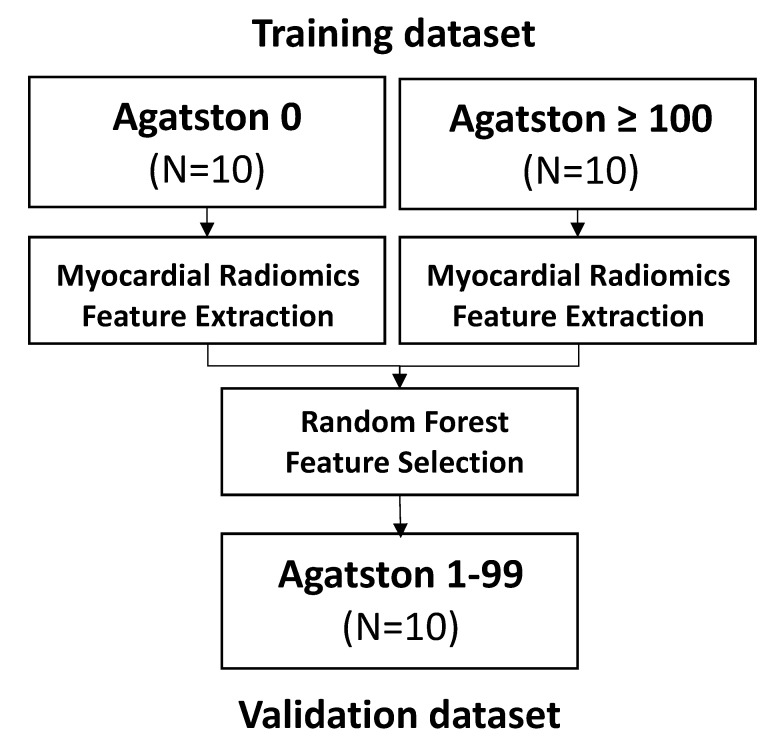
Consort flow diagram.

Non-enhanced-CT data were anonymized and exported from PACS. Plaque analysis was performed on axial nonenhanced scans with 2 mm slice thickness and Qr36 kernel using dedicated software (syngo.via, Siemens Healthcare GmbH, Forchheim, Germany). The study population was divided into different groups: As a training collective, patients with no sign of coronary calcification (Agatston score = 0) and patients with an Agatston score of above or equal to 100 were selected. Additional patients with an Agatston score of 1–99 were enrolled as an independent cohort for validation (Figure 1).

Window level and width were determined using the standard window-level setting from clinical routine.

**Figure 2:**
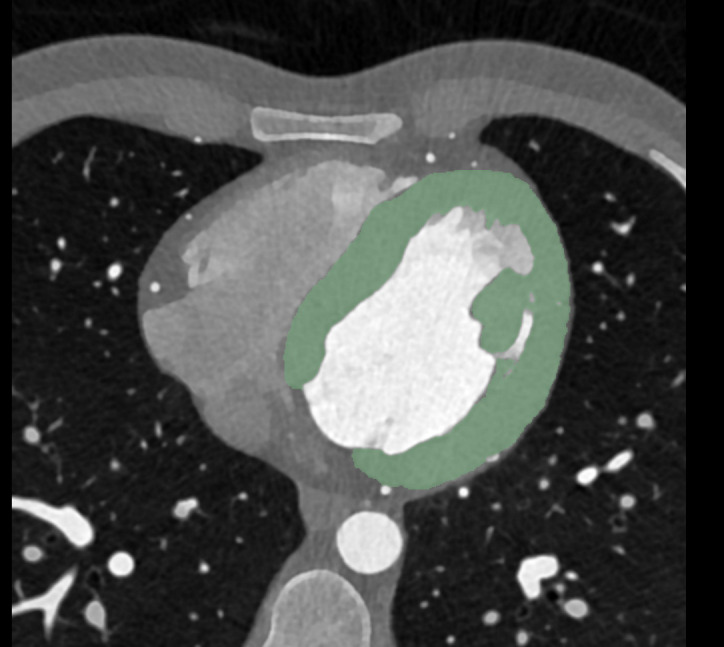
Segmentation of the left ventricular myocardium was performed on axial view with a slice thickness of 0.6 mm. An example case of a 21-year-old man is shown.

Additional axial images of contrast-enhanced CCTA were reconstructed with a slice thickness of 0.6 mm (increment of 0.4 mm) using a soft vascular kernel (Bv40). These data were also anonymized, exported, and stored in digital imaging and communications in medicine (DICOM) file format as well as converted into NIFTI file format for use with a dedicated segmentation tool (3D Slicer, Version 4.11) [25]. The whole left ventricular myocardium, including the trabecular structure and papillary muscle, was segmented semiautomatically by a radiologist with 9 years of experience in cardiovascular imaging. Figure 2 shows an example segmentation of the left ventricular myocardium in the axial view.

### 2.5. Radiomics Feature Extraction and Statistical Analysis

Radiomics feature extraction (first-order, shape, glcm, gldm, glrlm, gldm, glszm, and ngtdm) was performed in a dedicated software package (pyradiomics, Version 3.0.1) [26]. Extracted features were imported into statistical analysis software (R Statistics, Version 4.1.2, R Core Team, Vienna, Austria) [27] and assessed in RStudio (version 1.4.1717, Boston, MA) [28]. Mean and standard deviation values of quantitative parameters were calculated, and categorical variables were summarized as percentages. All radiomics features were normalized using the z-score:

z=((X−μ))/σ

Correlations of features were calculated as Pearson’s correlation coefficients. Feature visualization was performed as boxplots and heatmaps using the ComplexHeatmap Package in R; k-means clustering was performed. For feature selection, a permutation-based random forest (RF) classification was performed with the Boruta package for R.

## 3. Results

### 3.1. Cluster Analysis

**Figure 3:**
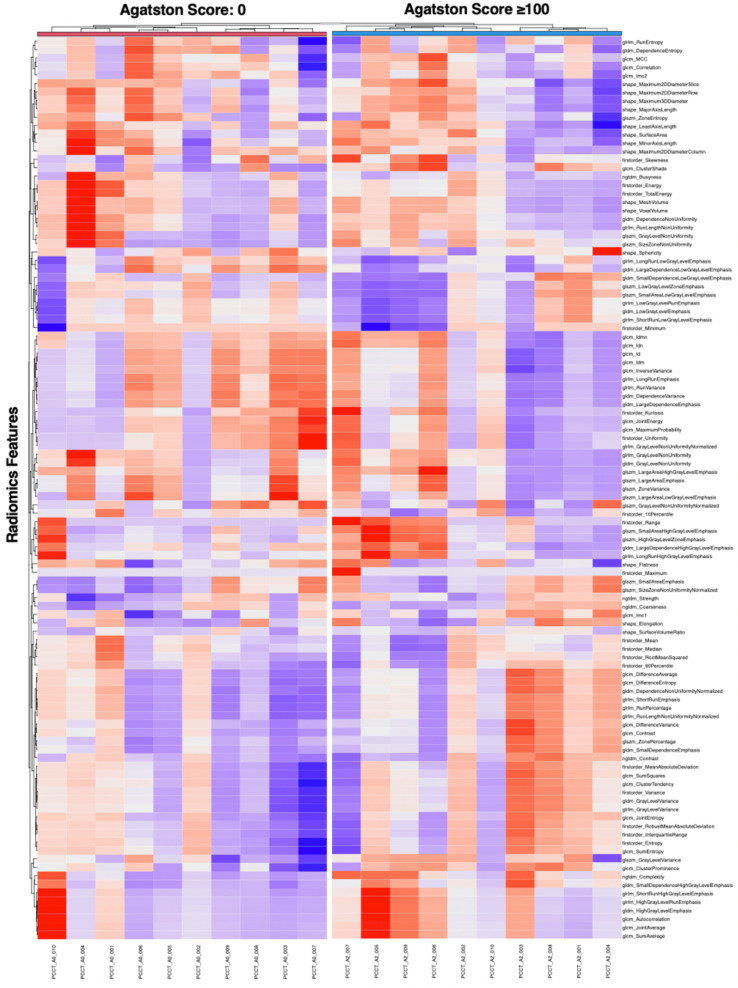
Unsupervised cluster heatmap of myocardial radiomics features.

After standardization, k-means clustering of radiomics features extracted from the myocardium of each patient was performed. Additional clustering within each Agatston severity group was added. These results are visualized in a heatmap (Figure 3).

### 3.2. Feature Selection

**Figure 4:**
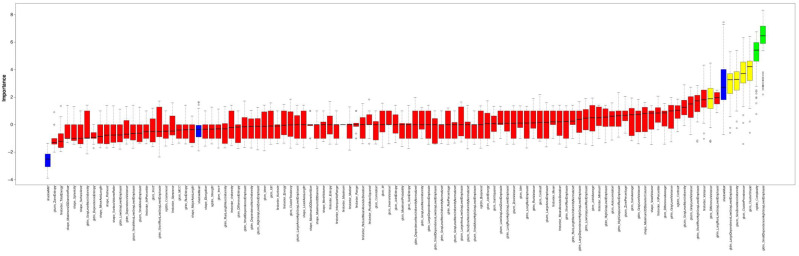
Random forest feature selection in 20 patients in training set.

RF feature selection was used for the selection of important features for differentiation of patients based on the left myocardial texture: First, random-forest-based feature selection was performed only on the patients with an Agatston score of 0 and an Agatston score ≥ 100. This led to the identification of “gldm SmallDependenceHighGrayLevelEmphasis”, “glcm ClusterShade”, “glrlm LongRunLowGrayLevelEmphasis”, and “ngtdm Complexity” as a feature set associated with a difference in Agatston score (Figure 4).

### 3.3. Internal Validation

**Figure 5:**
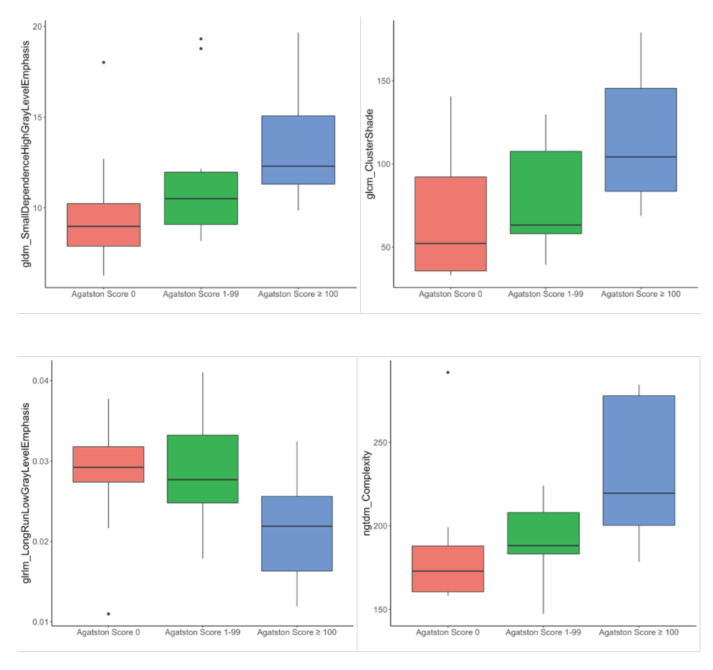
Distribution of “gldm SmallDependenceHighGrayLevelEmphasis”, “glcm ClusterShade”, “glrlm LongRunLowGrayLevelEmphasis”, and “ngtdm Complexity” feature within the dataset.

For internal validation, these radiomics features were investigated in the additional Agatston score 1–99 group. The respective radiomics scores are shown as boxplots in Figure 5 and summarized in Table 2.

**Figure 6:**
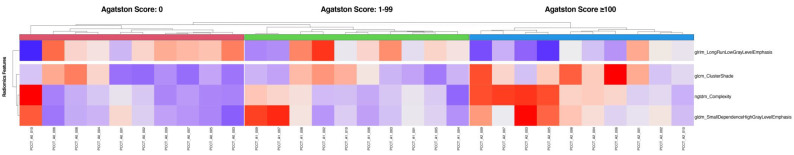
Unsupervised cluster heatmap of “gldm SmallDependenceHighGrayLevelEmphasis”, “glcm ClusterShade”, “glrlm LongRunLowGrayLevelEmphasis”, and “ngtdm Complexity” feature of 30 patients.

The Agatston score 1–99 group was settled between the two other groups, supporting the expected trend in the texture parameter changes of the left ventricular myocardium with an increasing Agatston score. In particular, gldm_SmallDependenceHighGrayLevelEmphasis showed mean values of 9.82, 11.79, and 13.44, respectively, in the subsequent Agatston groups (0/1–99/≥100). Similar distributions were shown for glcmClusterShade (68.42, 79.32, and 113.74), glrlm_LongRunLowGrayLevelEmphasis (0.0214, 0.0282, and 0.0283), and ngtdm_Complexity (185.12, 191.69, and 234.17). A combined heatmap of the reduced feature set for all Agatston severity grades is shown in Figure 6 (heatmap of all features for all patients, Supplementary Figure S1).

## 4. Discussion

In this pilot study, we demonstrated that the texture features of the left ventricular myocardium showed a possible association with the amount of coronary artery sclerosis. Differentiation between patients without coronary artery calcification and patients with coronary artery calcification was possible through four different radiomics-based texture parameters from the myocardium in this small sample size. Referring to the feature complexity, the value increased with increasing Agatston score, indicating a more heterogeneous structure. In line with this feature, cluster shade, as a measurement of skewness and uniformity, also increased with increasing calcification, outlining a greater asymmetry around the mean. This could be possible due to myocardial fibrosis resulting in a more heterogeneous texture structure. In detail, it was even evident that the amount of calcification tended to correlate with the severity of change in texture parameters, thus outlining a possible severity-associated effect of coronary artery calcification on the left ventricular myocardium with respect to the small sample size.

Nonenhanced coronary computed tomography allows a good estimation of coronary artery calcification, which can be classified into different risk scores by applying the in clinical routine commonly used Agatston score [29,30]. Hou et al. showed that CACS is not only capable of assessing the risk of major adverse cardiac events but also has an incremental and independent value compared with clinical risk factors [31]. Adding to this, Nicoll et al. found that the Agatston score is a more accurate predictor of significant coronary stenosis than conventional risk factors [32]. On the other hand, the absence of coronary artery calcification results in a significant reduction in major adverse cardiac events, but does not eliminate them [33].

Cetin et al. outlined different parameters derived from texture analysis in the myocardium of patients with different cardiovascular risk factors, including diabetes, hypertension, and cigarette smoking, acquired by late gadolinium enhancement sequences in MRI. Especially in the case of cigarette smoking, there was an increased heterogeneity in gray-level intensities, suggesting a diffuse process in the myocardium [34]. In the meantime, efforts are also increasing to visualize myocardial fibrosis in CT. Esposito et al. used texture analysis in late iodine-enhanced CT scans for evaluating the extracellular volume fractions (ECVs) in patients with recurrent ventricular tachycardia, with a good correlation of the texture parameter of general attenuation and central tendency with ECV and end-diastolic volume, as well as an inverse correlation with ejection fraction (EF) [35]. Acute or chronic myocardial infarction was detected by texture analysis in noncontrast, low-radiation-dose CT imaging on a second-generation, dual-source CT scanner by Mannil et al., especially outlining that this was invisible to the human eye alone. High-accuracy differentiation between healthy controls and patients with acute or chronic myocardial infarction was possible through certain texture features. However, the differentiation between patients with acute and chronic myocardial infarction was only moderately accurate but could be improved by pooling patients with both acute and chronic myocardial infarction (classification accuracy 86%). These results suggest a certain overlap of texture features in infarcts of different ages [36]. In contrast, Hinzpeter et al. illustrated the feasibility of texture analysis for distinguishing healthy from acutely infarcted myocardium with cardiac contrast-enhanced CT using a second-generation, dual-source CT scanner with a good to excellent intra- and inter-reader agreement for all first- and second-order features with different slice thicknesses [37]. Recently, Qin et al. visualized myocardial fibrosis by texture analysis in coronary artery CT by comparison with late gadolinium-enhancement sequences in patients with hypertrophic cardiomyopathy [38].

Nevertheless, myocardial texture analysis in cardiac CT is still in its infancy. Through the implementation of PCCT, a higher spatial resolution, as well as higher contrast-to-noise ratio and lower beam hardening artifacts, can be achieved [23,24], addressing the past limitations of radiomics analysis [39]. Furthermore, the distortion of features by beam-hardening artifacts can be excluded in radiomics texture maps. The first results showing the influence of PCCT in comparison with energy integrating detector CT on texture analysis of left ventricular myocardium outline comparable results in terms of first-order features, but differences in higher-order features, suggesting the possible impact of improved image quality on texture analysis [40].

Ultimately, however, this study also has limitations, where the small study population and the aspect of a single-center study must be emphasized. Additionally, this study made no reference to soft plaques, which can also lead to stenosing effects and thus influence the texture of the myocardium. Finally, no other risk factors, which could have contributed to texture differences of the myocardium such as tobacco use or hypertension, were considered in this study. Both issues should be considered in the future. Additionally, no comparison analysis with T1 mapping or late gadolinium-enhanced MRI sequences, or late iodine CT images was performed. This additional analysis for fibrosis detection is highly recommended for further studies. Yet, this pilot study is the first to investigate the myocardial changes associated with CACS in a photon-counting CT dataset.

## 5. Conclusions

In conclusion, this pilot study outlined the effect of coronary artery calcifications on texture analysis of the left ventricular myocardium, indicating a possible structural change in the myocardium depending on the Agatston score and a broader potential for myocardial image evaluation using computed tomography.

## Figures and Tables

**Table 1:** Patient overview. Mean and (SD) given for continuous variables.

	Overall	Agatston 0	Agatston 1–99	Agatston ≥ 100	*p*-Value
**Patient parameters**					
*n*	30	10	10	10	N/A
Age	58.27 (13.85)	50.6 (16,52)	63.2 (11.90)	61 (10.17)	0.091
Sex	22 male (73.3 %)	6 male (60.0 %)	7 male (70.0 %)	9 male (90.0%)	0.303
Stent	0	0	0	0	N/A
Agatston Score	270.10 (616,42)	0 (0)	29.74 (22.56)	789.57 (882.50)	0.002
**Scanner parameters**					
Tube voltage	120	120	120	120	N/A
Slice thickness	0.6 mm	0.6 mm	0.6 mm	0.6 mm	N/A
Kernel	Bv40	Bv40	Bv40	Bv40	N/A
Tube	Vectron ^®^	Vectron ^®^	Vectron ^®^	Vectron ^®^	N/A
Detector	PCD	PCD	PCD	PCD	N/A

**Table 2:** Higher-order radiomics features. Mean and (SD) given for continuous variables.

	Agatston 0	Agatston 1–99	Agatston ≥ 100	*p*-Value
gldm_SmallDependenceHighGrayLevelEmphasis	9.82 (3.39)	11.79 (4.03)	13.44 (3.21)	0.093
glcm_ClusterShade	67.42 (39.64)	79.34 (31.23)	113.74 (39.01)	0.025
glrlm_LongRunLowGrayLevelEmphasis	0.0214 (0.006)	0.0282 (0.007)	0.0283 (0.008)	0.062
ngtdm_Complexity	185.12 (40.14)	191.69 (22.34)	234.17 (41.94)	0.01

## Data Availability

The data presented in this study are available on request from the corresponding author.

## Supplementary Materials

The following supporting information can be downloaded at: https://www.mdpi.com/article/10.3390/diagnostics12071663/s1, Figure S1: overview of all radiomics features in training and validation group.
