# Supplementary figures and images for: Myocardial Radiomics Texture Features Associated with Increased Coronary Calcium Score—First Results of a Photon-Counting CT
Source: Diagnostics (Basel). 2022 Jul 8;12(7):1663. doi: 10.3390/diagnostics12071663 (PMC9320412; doi:10.3390/diagnostics12071663)

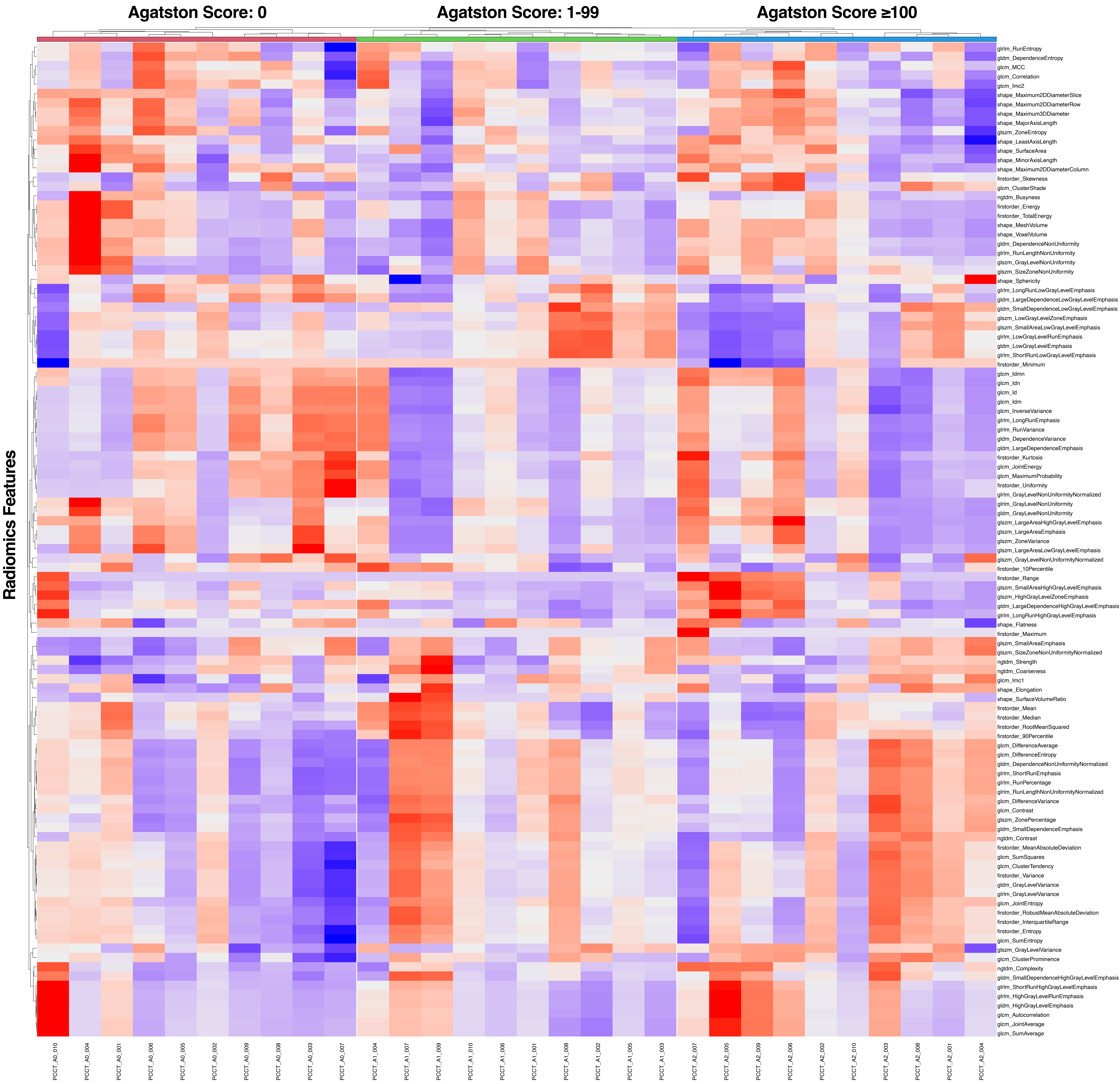

Supplement: Supplementary file 1 [file diagnostics-12-01663-s001.zip › diagnostics-1789404-supplementary.pdf]
